# Representations of ‘risky’ drinking during pregnancy on Mumsnet: A discourse analysis

**DOI:** 10.1111/dar.13948

**Published:** 2024-09-19

**Authors:** Lisa Schölin, Rachel Arkell

**Affiliations:** ^1^ Queens Medical Research Institute University of Edinburgh Edinburgh UK; ^2^ Centre for Reproductive Research & Communication British Pregnancy Advisory Service London UK

**Keywords:** alcohol, conception, discourse analysis, online forum, pregnancy

## Abstract

**Introduction:**

Online forums provide an environment for peer discussions to anonymously share experiences about sensitive topics. In this article we explore discussions about alcohol use during pregnancy, including representations of ‘appropriate’ behaviour and risks, in relation to alcohol use.

**Methods:**

We sampled Mumsnet posts from 2016 to 2021 and analysed these using a two‐staged approach: describing the content of original posts and employing discourse analysis on the entire thread which focused on unpacking the significance, activity and identity within the discourse.

**Results:**

Seventy‐three threads with 1554 replies analysed. Users engaged with different sources of evidence and mentioned guidelines and scientific sources, though most commonly provided, requested and appreciated anecdotal information. Risk was discussed in several ways but all users engaged with ‘othering’ drinking they perceived as risky. Only a few prescribed risks to any and all levels of drinking. There was a lack of knowledge around drinking while trying to conceive and early pregnancy. Expectations and norms of behaviour during pregnancy engaged in a ‘good motherhood’ discourse. Users positioned their drinking according to perceived norms through language choices; when not pregnant (or unknowingly pregnant) being ‘plastered’ was justified but when pregnant drinking only ‘sips’ or ‘tiny’ quantities of alcohol was considered appropriate.

**Discussion and Conclusions:**

Forum users demonstrated a desire to adhere to ‘responsible motherhood’, which did not equate to abstinence if consumption was perceived as acceptable and justified. Future research should explore information needs and effective approaches to health communication for pregnant women or those planning pregnancy.

## 1 INTRODUCTION

Alcohol exposure during pregnancy can lead to negative pregnancy and child outcomes, including foetal alcohol spectrum disorders (FASD) [1, 2, 3, 4]. However, no definitive risk threshold has been identified [3, 5] and establishing risks at low to moderate levels has particular challenges [6]. In the United Kingdom, recommendations state that the safest option for pregnant women and those who ‘could become’ pregnant is to not drink any alcohol [7]. The recommendation within the *Chief Medical Officers' (CMO) Low Risk Drinking Guidelines*, published in 2016, was intended to simplify the advice [8] and this abstinence‐based approach to alcohol and pregnancy is embedded within policy frameworks for health professionals in the United Kingdom [9, 10]. Subsequent research has, however, acknowledged that women's interpretation of risks related to lifestyle behaviours provided by health‐care providers and through public health messaging in guidelines is complex and may require a layered approach to risk communication [11]. Indeed, the precautionary principle is often deployed in the absence of data on safety and harm [12] and the change in the CMO guidelines has been described as ‘value‐based rather than evidence‐led’ [13]. Interpreting uncertainty as inherently risky can result in a ‘state of hypervigilance’ for pregnant women as ‘the boundaries between “dangerous” and “safe” and between “reckless” and “responsible” are shaped in capricious and rigid ways’ [14, p. 38].

An important question relating to changes in public health guidelines is the impact it has on knowledge and behaviour. In the United Kingdom, a general population survey conducted 1 month after the announcement of the CMO guidelines in 2016 found that 71% were aware of the new guidelines but only 8% knew their content [15]. Furthermore, the changes did not align with professional bodies that provide practice guidelines, leading to conflicting advice being available and relied upon by midwives [16]. Similarly, more recent research from Australia, where abstinence‐based guidance has been in place since 2009, showed 64% of pregnant women surveyed in 2017–2018 were aware of guidelines related to pregnancy and alcohol and of those, 78% knew the recommendation was abstinence. The study did not find any significant difference in knowledge between those who continued or stopped drinking during pregnancy [15]. A study from Denmark concluded that changes in guidelines did not necessarily have an impact on alcohol consumption, attitudes or knowledge towards alcohol use during pregnancy [17]. While the authors noted that the guidelines were not implemented comprehensively among health professionals, they found that there has been a decreasing trend in prenatal alcohol consumption, including when guidelines were expanded to include some level of maximum consumption rather than abstinence. Knowledge about existing guidelines may, therefore, not be universal nor necessarily impact on behaviour. Changes in norms around prenatal alcohol use, and changes in messaging in, for example, mass media to promote abstinence [18] may also contribute to shaping behaviour and attitudes.

A range of factors influence why women may continue to drink during pregnancy. Popova et al. [19] found that women held beliefs that alcohol had benefits to foetal and maternal health and that there was no risk when consumed in small or moderate amounts, or when limited to special occasions. Individual behaviours were, however, connected to cultural norms, social acceptability and cultural practices or expectations to consume alcohol [19]. This was further emphasised by Taylor et al.'s. [20] meta‐ethnography focusing on women who drank at high levels during pregnancy. Women reported feeling concerned about their drinking but were reassured by the fact that the people around them are also engaging in the same behaviour. The authors also highlighted that when considering knowledge of risk, studies have tended to focus on internal processes (cognitive dissonance theory) rather than also considering wider socio‐cultural factors that may influence both knowledge and behaviour. They concluded that prenatal alcohol use is influenced by perceptions about norms of behaviour in which women adhere to behaviours which fit with perceptions of being a ‘good mother’, which are specific to their own cultural context [20, p. 2].

A core component of western, contemporary ideals of ‘good motherhood’ is a state of hypervigilance towards any risk, however, remote, in pregnancy [21, 22]. The pregnant body has been identified as a potential site for risk [23], with pregnant women being regarded as the ‘potentially dangerous vessel’ which poses this risk herself [24]. This level of individualisation of risk is often reinforced by medical advice and parenting/pregnancy manuals [25] and results in a demand for complete risk elimination even to the detriment of the pregnant woman herself [14]. For behaviours which are seen as intrinsically risky, such as alcohol use, risk management often furthers a moral dimension [26]. This has been explored in the context of media and public health reaction to maternal behaviours that have facets of a moral panic, including prenatal alcohol use, second hand smoking and obesity [21]. However, alcohol is arguably viewed differently because of its role in social contexts. Alcohol use during pregnancy has historically, specifically within a US context, been viewed through a lens of moral panic and necessitating the need for government interventions and social control [26]. As such, some argue that social framing may be more influential than medical knowledge in defining what amounts to ‘risky’ behaviour [23, 25]. As Burton‐Jeangros notes: ‘pregnancy constitutes an effective case study in the sense that expectations about medically recommended behaviours cumulate with expectations about motherhood’ [23, p. 433]. Pregnant women, therefore, interpret and construct risk within these moral frameworks alongside evidence and knowledge of medical risks.

Within a broader scholarship of motherhood, the study of ‘motherhood online’ has developed as a discipline within research and discourse analysis on pregnancy and parenting [27]. Previous studies have explored issues such as perinatal depression [28] and vaping during pregnancy [29]. However, few studies have explored discussions about alcohol use during pregnancy [30, 31]. On the backdrop of changes in guidelines in the United Kingdom and the wider factors influencing women's perceptions of risk this study aimed to explore risk perceptions within online discussions about alcohol use during pregnancy following the publication of the CMO guidelines in 2016. Online forums are an alternative data source to study discussions about a topic, such as alcohol, where an anonymous environment may allow individuals to speak more openly about their experiences and opinions [32].

## 2 METHODS

We undertook a qualitative study to explore discussions about alcohol use during pregnancy in an online forum space, looking to firstly describe *what* was discussed and *how* it was discussed. We used an abductive approach informed by a rich sociological literature on risk management in pregnancy [21, 22, 23, 24, 25, 26, 33]. An abductive approach ‘requires in‐depth familiarity with a broad array of social theories and an intensive engagement with observations in order to develop theoretical observations’ [34, p. 1]. As Tavory and Timmermans suggest, an abductive approach, rooted in pragmatism, differs from inductive and deductive approaches as it involves a deep engagement with a ‘back and forth’ process between data and theory to generate new ideas, rather than a ‘one‐way’ inductive or deductive approach [35]. Hybrid inductive/deductive approaches, such as an abductive approach, strives for more equal engagement with theory and data. This approach enabled us to draw on our in‐depth knowledge of health policy, risk communication, and contemporary parenting ideologies to make sense of the observations within threads. We chose discourse analysis as the most appropriate methodology to answer the aim of our study; discourse analysis allows researchers to interrogate how and why language is used in any given time and place [36]. Using Gee's [36] approach to discourse analysis allowed us to explore how forum users (from hereon ‘users’) built identities and enacted practices and how they recognised the identities and practices that others were building around them.

### 2.1 Sampling strategy

This study draws on data collected from a sample of threads posted on Mumsnet (a UK online parenting forum). We selected it as it is the top‐rated site with the most forum users across all online support forums for pregnancy and parenthood in the United Kingdom [37]. Using the keyword ‘alcohol’, threads were identified within the conversation topics ‘pregnancy’ and ‘conception’ between 1 January 2016 and 31 December 2021. Our baseline for the sampling frame was the announcement of the CMO guidelines and to cover key developments in United Kingdom in the years since these guidelines were published. For the 6‐year period there were 438 threads posted, which was unfeasible to analyse in its entirety within the time frame of the project. A sampling frame was therefore applied in which threads posted in January, March, May, July, September and December each year were screened for inclusion, with the aim to screen at least 50% of all threads for each year. This allowed us to cover months mapping onto key events (e.g., release of The Guidelines in January 216) and months where social drinking in the United Kingdom is common (such as Christmas). Years where these months did not result in at least 50% of all threads oversampling was employed, leaving 271 threads to screen for eligibility. The eligibility criteria were: (i) threads including advice on current/planned consumption during pregnancy; (ii) discussions on safe amounts of alcohol that can be consumed; (iii) discussions about harmful effects from alcohol exposure; or (iv) discussions about official drinking guidelines or advice from health‐care providers or others.

### 2.2 Data analysis

All eligible threads were saved as PDFs and imported to NVIVO for analysis. For the first part of our analysis, we coded the content of each original post that started the thread (purpose of the post, topic, number of days the discussion went on for and number of replies). We summarised this using descriptive statistics.

For the second part of the analysis, informed by Gee's [36] approach to discourse analysis, we focused on unpicking the (i) *significance* placed on an issue; (ii) *activity* of the discourse; and (iii) *identity* is the discourse portraying. In outlining an ideal discourse analysis Gee [36] includes another four building tasks (relationships, politics, connections and sign systems and knowledge), though acknowledging that ‘any real discourse analysis only deals with some the questions’ (p. 141). Focusing on these three building tasks and questions was a pragmatic decision, primarily owing to the size of our project and relevance to our research question. Our methodological and analytical choices allowed us to explore the interplay between ‘official’/expert guidance and personal experience, and how continued conflict around alcohol and pregnancy is negotiated within an online space. Initial coding was conducted on seven threads, which were double coded by both Lisa Schölin and Rachel Arkell. An initial coding framework was developed using these threads and in‐depth discussions were held to resolve any discrepancies and to ensure consistency in the coding. After the initial pilot coding, the coding framework was finalised and applied to the remainder of the threads, split between Lisa Schölin and Rachel Arkell. The final coding framework is included in Table S1, Supporting Information.

Discussions about interpretation of discourse, however, continued throughout the independent coding process. These discussions were essential as the two authors have different background and areas of expertise (Lisa Schölin: public health and alcohol research; Rachel Arkell: law and medical ethics). While there was a shared understanding in terms of the theoretical framework and evidence based which underpinned the abductive analytical approach, our different backgrounds influenced our initial interpretation of discourse whereby coding discussions allowed us to develop a shared interpretation and expand on each other's approach to the data.

### 2.3 Ethical considerations

Conducting research on online platforms is associated with several ethical issues. The British Psychological Society has set out four key principles for internet‐mediated research: ‘respect for autonomy, privacy and dignity of individuals and communities; scientific integrity; social responsibility; and maximising benefit and minimising harm’ [38, p. 7]. The British Psychological Society guidance and its principles for internet‐mediated research were followed and therefore only threads that were publicly accessible (i.e., did not require a profile to view the thread) were included. Aligning with previous work, no usernames of the users were included [39, 40] and no verbatim quotes [41] are included in this paper. Instead, we only present keywords or key phrases that were used in the threads, which when cited verbatim are denoted with. This allowed further anonymisation of user data and prevents identification of users by Google searching verbatim quotes. Ethical approval was granted from the Edinburgh Medical School Research Ethics Committee (22‐EMREC‐056).

## 3 RESULTS

### 3.1 Overview of original posts

For the first part of our analysis, we provide a summary of the characteristics of the posts which started the threads is provided in Table 1. Within the 73 threads there were 1554 replies to the original posts. The topics within the original posts in the sample were around drinking before knowing about the pregnancy or drinking while trying to conceive (TTC) (including in the ‘two‐week wait’ between date of conception and confirming pregnancy), and no‐ and low‐alcohol (NoLo) products. While responses *within* threads discussed drinking while knowingly pregnant, only six original posts covered this topic. Users were most commonly seeking advice or reassurance regarding the topic of the original post.

**TABLE 1:** Characteristics of original posts.

Category	Grouping	*N*	%
Purpose	Advice seeking	24	33
	Seeking opinions/experiences	21	29
	Seeking reassurance	14	19
	Seeking advice/reassurance/experience and information/airing frustrations	5	7
	Information seeking	5	7
	Airing frustrations/confessions	3	4
Topic	Exposure before knowing	25	34
	Drinking while trying to conceive	23	32
	Low/no alcohol	10	14
	Other^a^	7	10
	Consumption in pregnancy	6	8
	Addiction	2	3

^a^ Food containing alcohol, struggling not to drink, high exposure before knowing, craving a drink, accidental exposure, relapse of alcohol dependence and drinking until finding out.

Three themes were developed from the discourse analysis: importance of evidence, users' perceptions of ‘risky’ drinking, and how users differentiated between ‘risky’ drinking and understandable exposure to the foetus. These themes are outlined in the following sections.

### 3.2 The importance given to different kinds of evidence

#### 3.2.1 Official guidelines

Many users highlighted changes over time in public health guidance and attributed the source of current guidance to ‘the NHS [National Health Service]’. The tone ranged from supportive to ‘hostile’, as some felt that there is a lack of evidence around harm from small amounts of alcohol and that this is not reflected in the guidance. Two separate stances were found. Within the first, users felt the precautionary approach is appropriate and reflects a lack of certainty about risk. Those supportive of approach felt that providing advice to keep alcohol at low levels would mean people would ‘push the limits’ while some had a more neutral stance considering the guidance ‘reasonable’ and ‘erring on the side of caution’. The choice in language among these users was mainly in a matter‐of‐fact way without any moral undertones. Within the other stance, users were overtly hostile and argued it removes autonomy from women to weigh up the risks, branding it ‘unhelpful’, ‘scaremongering’, and that it was because women are viewed as ‘too stupid’ to drink within ‘safe’ (previous guidelines) limits. On occasion, these opposing views clashed where some users defended their opinion that they felt the guidance was ‘for a reason’, with judgemental responses, such in one instance in which a user was described as ‘sanctimonious’ for promoting the abstinence advice. The knowledge of older guidance was used by some users to justify drinking any alcohol while pregnant and some users perceived there to be no/low risk since older guidance ‘allowed’ for some alcohol.

#### 3.2.2 Academic research

There was a mixed perception of what the academic research has found in relation to risk of harm. A few users spoke specifically about study design being an issue with establishing any risk threshold, but most spoke in general terms about research. Some users were unsure about the evidence, though many were speaking with conviction about their position and perception of risk. This worked to enhance the interpretation of risk, mainly through informing, advising and reassuring users who were concerned or just unsure about the evidence where lack of evidence of harm at low levels was often communicated. Though there were both arguments that there is ‘no evidence’ that small amounts harm the baby and that there is ‘lots of research’ showing alcohol harms the baby even at low levels. Users presented themselves as able to evaluate information they had encountered and with several emphasising they had done ‘their own research’ and positioning themselves not as passive recipients of information. The book *Expecting Better*, by American economist Emily Oster, was frequently mentioned and discussed in positive terms, described as ‘well researched’, ‘sensible’, and going into ‘all the statistics of risk’ and the accuracy of the content in the book was only questioned in one instance.

#### 3.2.3 Anecdotal evidence

Anecdotal evidence was the most discussed source of evidence and was considered valuable, especially when users were anxious about unintentional exposure. When sharing anecdotal evidence, users positioned themselves as responsible and informed mothers in the way they expressed concerns or how they reassured other. This evidence primarily focused on exposure while TTC and in early pregnancy. A few extreme cases included women who were not TTC and had discovered the pregnancy at a late stage and were concerned about sustained exposure. Users with unintentional exposure described feeling guilt and anxiety, using language such as ‘hating myself’, which tended to be responded to through reassurance using positive outcomes from similar experiences where children who had been exposed are ‘bright and bouncy’, ‘perfect’ or ‘advanced’. A few users utilised more humoristic language, such as one user noting her baby was not born with ‘two heads’ despite exposing them to wine. The discourse surrounding anecdotal evidence mainly put significance on the belief that many women have had unintentional exposure without negative outcomes. There were only a few exceptions where anecdotal evidence was questioned as valid and applicable to other people.

#### 3.2.4 Health professionals

Health professionals were seen as having the ‘correct’ knowledge and able to support the users' concerns. Seeking support from a midwife or general practitioner was actively encouraged in almost all instances where users were worried about exposure, except in one case of very low‐level exposure where other users said it would be inappropriate to contact the general practitioner to ask for advice. There were several accounts of health professionals advising that low level alcohol use was not harmful in which users' language was authoritative and certain, informing other users that their health professional had said there was ‘zero chance’ of harm or ‘laughing’ at the suggestion that small amounts were risky. There was a mixture between reassuring, by using midwives' or doctors' advice about small amounts being fine, and advising, by directing worried or anxious users to be honest and seek advice from a health professional. This discourse further enhanced the creation of an identity of a responsible mother by, regardless of what the advice was, actively seeking out information from an authoritative source. Users were often reassured that it was unlikely that they needed to worry but health professional advice was recommended to put any concerns at ease. Reassurance was often provided through differentiating concerned users from women perceived to be at risk of having a child with FASD (see Heavy drinking—defining the ‘others’).

### 3.3 Unpicking how forum users represent ‘risky’ drinking

#### 3.3.1 Minimising the risk of harm

Within this discourse users emphasised levels of exposure that they considered non‐risky. However, what they considered risky alcohol use was rarely defined in specific quantity/frequency. Words or phrases used to describe non‐risky drinking were ‘small amounts’, ‘one‐off’ or ‘odd’ instances. The main function was to provide reassurance to users who disclosed alcohol consumption, including what we call *conditional reassurance* in which users were told their small and/or infrequent consumption would not have caused harm provided they either abstained from hereon or continued to consume ‘acceptable’ small amounts. Another function, albeit less frequent, was to ‘combat’ claims that any amount of alcohol, including single drinks, in pregnancy can cause harm. Here, some users expressed their disbelief in a causal relationship between low levels and harm. In allied posts, emphasis was placed on choice and individual attitudes towards risk, furthering the identity of an autonomous person. These users stressed their ability to rationalise risk, without suggesting the need to impose their choice on anyone else.

#### 3.3.2 Maximising risk

Some users felt that ‘drinking heavily’ was not the only consumption that could cause ‘FAS’ or ‘varying degree of FAS’. Some explicitly described alcohol as a ‘poison’ or ‘teratogen’ and risk avoidance became the solution. Users engaging with this discourse felt that drinking was an ‘unnecessary risk’, which would be ‘doing harm’ or ‘betraying the baby’. Others positioned the foetus as vulnerable because their ‘tiny livers’ cannot metabolise alcohol. Users explained how they ‘could never’ take the risk of exposing this vulnerable being to alcohol, claiming those who do are prioritising their own ‘pleasure’ or ‘selfish self‐gain’. In a small number of posts explicitly morally laden language was used to characterise and represent women who drink as irresponsible and careless. Some users used *perceived equivalency*, a rhetorical tool where consuming alcohol in pregnancy was compared to giving a newborn a glass of wine. This maximised the risk of any alcohol use during pregnancy but this was contested by other users who considered it inaccurate. Within this discourse, the ‘good motherhood’ identity was emphasised through the perceived need of acting responsibly. Those with experience of knowing or working with children with FASD felt that pregnant women would not drink if they knew what the outcomes could be. The ‘good motherhood’ identity was also portrayed among users who had unintentional exposure and engaged in self‐criticism, describing feeling ‘worried’ or ‘petrified’ to demonstrate that their behaviour was not intentional, nor desirable.

#### 3.3.3 Heavy drinking—Defining the ‘others’

Many users considered, implicitly or explicitly, that heavy drinking was associated with risk and clearly distinguished from occasional or low‐level consumption. Common language included ‘binge‐drinking’, ‘continual exposure’ and ‘alcoholics’. Users also enhanced this view by referring to stronger types of alcohol in conjunction with quantity perceived as harmful, such as drinking ‘a bottle of vodka’ per day. FASD was described as resulting from ‘heavy’ or ‘sustained drinking’ (apart from a few instances where it was associated with other levels of drinking, see ‘Maximising harm’). Users engaged in this discourse wanting to inform others and spoke with authority which did not seem rooted in anything further than the poster's own confidence in their own beliefs, apart from a smaller number of users who cited a professional identity (e.g., midwife) or relaying informed they had obtained from a health‐care professional. Within this discourse, users created identities that distanced women with alcohol problems from users of the forum. Even women who were concerned about heavy unintentional exposure were represented as different to the women users perceived to engage in risky drinking. Notably, no posts included self‐disclosure of alcohol problems during pregnancy, though in one post a user described how their sister had relapsed while pregnant and one user who self‐described as an ‘alcoholic’ was seeking advice on drinking until finding out as they had relapsed before finding out.

### 3.4 The differentiation between ‘risky’ drinking and ‘understandable’ exposure

#### 3.4.1 Justifying any exposure during pregnancy

This discourse mostly focused on unintentional exposure during early stages of pregnancy, in which users emphasised they would have behaved differently had they known, using language such as ‘in my defence’. Users commonly stated that many women drink before they know they are pregnant, which worked to provide reassurance and to create shared experiences and help alleviate feelings of regret or remorse. Where users expressed uncertainty of how whether alcohol could be consumed while TTC, many responded abstaining was not necessary during this period and to ‘drink until it's pink’ (positive pregnancy test). Users who had chosen to consume alcohol during pregnancy described consuming ‘sips’ or ‘gulps’, indicating they perceived this as low risk and within ‘appropriate’ behaviour (see ‘Minimising risk’). This language was distinctly different to descriptions of normative alcohol use when not pregnant, such as drinking ‘huge’ amounts or making light of heavy drinking with a comical undertone of being ‘hammered’, ‘trollied’ or ‘piddled’. Generally, the way users described and justified drinking during pregnancy (either when knowingly pregnant or before finding out) engaged with an identity of someone who is ‘following the rules’. Drinking was particularly justified at certain occasions such as hen parties, holidays, or Christmas and many justified more than once why they had consumed alcohol, with only a few explicitly only justifying drinking while knowingly pregnant because they ‘fancied it’. Users distanced themselves from what they perceived as ‘risky drinking’, by justifying the small amount, the unintentional nature of the exposure, and/or that it had occurred at a special occasion in which alcohol use is normal and expected. Some users who had chosen to drink during pregnancy distanced themselves from those advocating abstinence by using sarcastic or judgmental language such as ‘shock horror’ when disclosing consuming alcohol or calling those advocating abstinence ‘sanctimonious’. A smaller number of posts simply referred to the choice as being ‘personal’ or a ‘woman's right’ which had a more defensive tone as the users might have expected judgement about their choices.

#### 3.4.2 Cravings

Cravings related to social norms around drinking behaviour and often presented in hyperbolic language. Users described feeling like ‘murdering’ a glass of wine or ‘dying’ for a drink but stated or implied they were abstaining from alcohol. Some posts used a range of descriptors to create a shared experience related to drinking alcohol, such as using adjectives like ‘cold’ or ‘crisp’ which demonstrated a shared knowledge of what makes an alcoholic drink enjoyable for some people. Furthermore, this created an understanding of why this craving, within given context where abstinence was expected, was acceptable. Users also emphasised/justified the context of the craving (e.g., a special occasion) to ensure other users understood their emotional response. This further bolster the ‘good motherhood’ identity as users may have felt like they needed to justify their alcohol cravings to enact this identity, particularly given the social expectations of abstinence.

## 4 DISCUSSION

This discourse analysis of online discussions adds to the existing literature on how ‘good motherhood’ and social norms around alcohol use influence interpretation and conceptualisation of risks associated with drinking during pregnancy and the pre‐conception period. Furthermore, the study adds to a currently limited literature on what impact drinking guidelines may have had on attitudes or behaviour towards drinking during pregnancy.

Users enacted and relied on different identities which speak to how they want to be perceived by others. The majority, if not all, of these representations rely on western ideas of maternal responsibility—and users are keen to ‘prove’ that they are responsible, ‘good’ mothers with regards to their approach to alcohol in pregnancy. This can be interpreted as a signal moment, whereby a pregnant person's attitude and conceptualisation of drinking alcohol in pregnancy are moments at which a woman's ‘fitness to parent’ will be judged [42]. This was evident in the ways users positioned themselves as making informed decisions and adhering to their perception of appropriate behaviour (drinking small amounts or by abstaining). Both these narratives signalled that users were expecting judgement as the idea that any amount of alcohol could harm the developing foetus result in concerns among women that they're going to be ‘seen as a “bad” mother’ [33]. This view is largely shared by intensive [43] or total [44] models of motherhood, which strive for complete risk elimination, starting in pregnancy and increasingly in the pre‐conception period [45]. The decision to abstain from alcohol, therefore avoiding any risk, is a marker of responsible behaviour. This was evident in the way some forum users felt that consuming alcohol would be ‘betraying the baby’, representing behaviours that would be considered antithetical to conceptions of ‘good motherhood’. Our findings did, however, reveal a strong subversion of this specific depiction of ‘a good mother as one who abstains’ but this again relies on the ‘responsible mother’ as a key identity. Users spoke at length at their ability to rationalise risk and make the ‘right’ decisions for themselves and their pregnancies. For some, this may involve drinking at more ‘acceptable’ (i.e., low) levels of alcohol during pregnancy [19] and for others, abstinence was the only ‘correct’ choice. Despite differing approaches to consumption, both groups enacted and relied on identifies of ‘responsible’ and therefore, ‘good’ mothers. Similar views have been reported in a study including face‐to‐face interviews with 40 Australian women, who described that the pressure they felt to adhere to social norms around what appropriate behaviour among expectant mothers were, influenced them to abstain [46].

Coming to the issue of the CMO guidelines, our findings suggest that interpreting the guidelines is complex and users demonstrated knowledge gaps in when alcohol may be harmful. This aligns with the conclusion by Taylor et al. [20] that ‘women take a more nuanced approach to weighing up the risks and benefits of drinking during pregnancy and do not always respond to messaging as intended’ (p. 9). This was also described in a study of views around alcohol use in pregnancy among 46 Swiss couples, which found that understandings of risk are complex and ‘situated in the context of everyday life and the embodied experience of the risk’ [47, p. 8]. Some scholars have argued that the way uncertainty in the evidence of risk thresholds has been framed implies that uncertainty is associated with risk [33]. Our findings suggest that in the time since the guidelines were introduced, there was not a ‘simple’ interpretation of the abstinence message, as those who felt low level consumption was not risky could justify it, and often could get confirmation or reassurance from others that this level was not considered risky. This also includes health professionals, as we observed users recounting occasions in which health professionals had ensured them that consuming small amounts was not harmful. Furthermore, the pre‐conception or around‐conception consumption was not interpreted simply either and concerns about consumption in early stages were addressed by others by suggesting it would be ‘risk free’ to keep drinking until a positive pregnancy test. The common nature of drinking in the TTC period, which caused many users to feel anxious, suggests that there is a need for further exploration of women's interpretation of this part of the CMO guidelines. Such interpretations need to take into consideration that information and recommendations have changed over time and may influence attitudes, rather than only focusing on psychological processes or ‘misinformation’ [20]. The shift towards a precautionary approach during the TTC period as a ‘value‐based’ decision rather than an ‘evidence‐led’ one [12] can make informed choice difficult for those planning a pregnancy.

Overall, the way users positioned themselves reveal two important findings. First, the view of the choice to drink during pregnancy is socially conditioned, whereby users distanced themselves from ‘risky’/’problematic’ drinkers. Those considered at risk of negative pregnancy and/or child outcomes were those dependent on alcohol, at times labelled ‘alcoholics’ and this active linguistic choice may be rooted in or attributed to stigma. Even those who do drink, described consuming small quantities, or those had drunk to excess had done so unintentionally or performed great remorse and deeply strive to be perceived as doing the ‘right’ thing. Hammer et al. [47] also found that women who drank occasionally described, as we also found, ‘sips’ or ‘just half a glass’ with further justification of special occasions. Other women expressed guilt even from small amounts of alcohol which they related to taking risk and being a ‘bad mother’ [47]. The inherited desire among women of wanting to make the best choices possible for their children, choices which they make drawing on a host of internal and external factors [19, 20, 47], should guide development of supportive messages that seek to empower and reassure women to make informed decisions without potentially creating anxiety or stress. There is also a need to further study how the public health advice on alcohol and pregnancy is best communicated in a way that provides sufficient evidence and information around why the guidance is to abstain. Second, crucially, the official guidance is almost moot for a lot of users. This may be particularly relevant where users acknowledged lack of evidence for harm at low levels of alcohol. This reflects similar findings from a German forum study where users critiqued complete abstinence recommendations with explicit notions on the need for explanations of risk at different levels of consumption, as opposed to what they felt was a ‘complete ban’ on alcohol use [31]. As discussed in our findings, often the most sought after—and valuable (particularly in terms of reassurance)—evidence is anecdotal. When making decisions around pregnancy, women draw on a range of ‘evidence’ to guide them with how best to proceed [20]. Our findings, which do point to a hierarchy of evidence in terms of which type (anecdotal, health professionals, academic studies, ‘official’ guidance) were more often sought, are consistent with existing views that women make decisions in pregnancy based on a range of evidence [48], including the experiences of their families and partners, and ‘their cultural societal norms and expectations.’

Finally, reflecting on Gee's [36] approach to ensuring validity in a discourse analysis, in which he acknowledges that convergence and linguistic details are most immediately key, we note that there was convergence across themes in that users wanted to appear responsible to peers. This was reflected in their desire and value of reassurance, partly through their linguistic choices to either minimising or maximising risk. In addition to reassurance these linguistic choices across the analysis were made to justify ‘acceptable’ alcohol exposure. Our findings indicate agreement, to some extent, with other studies showing how users believed that abstinence was an indicator of ‘good motherhood’ [31]. Our findings, however, also indicate a desire to adhere to ideals of ‘good motherhood’ even when some alcohol (intentional or not) had been consumed.

### 4.1 Strengths and limitations

The strengths of this study include the structured sampling framework and large number of posts analysed, resulting in conclusions drawn upon the opinions of a large number of individuals. However, there are limitations that need to be acknowledged. First, the sampling frame did not include all potentially relevant posts over the period and information within months that were not sample may have differed or added additional insights. Secondly, a ‘full’ discourse analysis would include the remaining four of Gee's building tasks and other aspects of the discourse remains unexplored. Thirdly, we did not explore whether there was a shift in the discourse over time. as it was not within the scope of the research question, and language and perceptions may have shifted from just after the change in guidelines to later on in our data collection period. Finally, while online discussions may be honest within the guise of anonymity these views may not represent pregnant women or women TTC more broadly. Other researchers have highlighted that there is a lack of data on socio‐demographic characteristics of Mumsnet users limiting any meaningful conclusions as to representativeness [41]. Due to the perceived need to adhere to ‘good motherhood’, individuals who drink at levels users described as being risky might not have felt able to engage in the discussion and put forward their opinions.

## 5 CONCLUSIONS

Interpretation of the message to abstain from alcohol when pregnant or TTC varies and the target group does not appear homogenous. Normative drinking behaviours including binge drinking, that may occur around conception can result in unintended exposure even when forum users were monitoring their fertility. Users on this forum mainly dismissed unintended exposure around conception as harmful. There is a prevailing perception that low levels of alcohol are not harmful which was further demonstrated and articulated by justifying both amounts and types of alcohol consumed and the occasion on which it was consumed. Through exploring the choice and use of language on the forum a central ‘good motherhood’ discourse was evident. While users relied on some ‘official’ evidence to guide their perceptions about what was ‘correct’ behaviour during pregnancy, anecdotal evidence from others was rated highly but health professionals were also seen as appropriate sources of information. Future research and development of public health messaging should explore how to accurately balance information about risk and harm with the strong desire of women to act in the best interest of their future children.

## AUTHOR CONTRIBUTIONS

Each author certifies that their contribution to this work meets the standards of the International Committee of Medical Journal Editors.

## CONFLICT OF INTEREST STATEMENT

The authors report no conflicts of interest.

## Supporting information


**Table S1.** Coding framework.

## Data Availability

The data can be downloaded from Mumsnet or can be provided from the authors upon request.

## References

[1] Sundermann AC , Zhao S , Young CL , Lam L , Jones SH , Velez Edwards DR , et al. Alcohol use in pregnancy and miscarriage: a systematic review and meta‐analysis. Alcohol Clin Exp Res. 2019;43:1606–1616. 31194258 10.1111/acer.14124 PMC6677630

[2] Patra J , Bakker R , Irving H , Jaddoe VWV , Malini S , Rehm J . Dose‐response relationship between alcohol consumption before and during pregnancy and the risks of low birthweight, preterm birth and small for gestational age (SGA)‐a systematic review and meta‐analyses. BJOG. 2011;118:1411–1421. 21729235 10.1111/j.1471-0528.2011.03050.x PMC3394156

[3] Mamluk L , Edwards HB , Savović J , Leach V , Jones T , Moore THM , et al. Low alcohol consumption and pregnancy and childhood outcomes: time to change guidelines indicating apparently ‘safe’ levels of alcohol during pregnancy? A systematic review and meta‐analyses. BMJ Open. 2017;7:e015410. 10.1136/bmjopen-2016-015410 PMC5642770 28775124

[4] Riley EP , Infante MA , Warren KR . Fetal alcohol spectrum disorders: an overview. Neuropsychol Rev. 2011;21:73–80. 21499711 10.1007/s11065-011-9166-x PMC3779274

[5] Henderson J , Gray R , Brocklehurst P . Systematic review of effects of low‐moderate prenatal alcohol exposure on pregnancy outcome. BJOG. 2007;114:243–252. 17233797 10.1111/j.1471-0528.2006.01163.x

[6] Whitfield JB . Commentary: causation versus association for fetal effects of maternal alcohol use. Int J Epidemiol. 2020;49:1995–1997. 10.1093/ije/dyaa086 32617572

[7] Department of Health . UK Chief Medical Officers' Low Risk Drinking Guidelines. 2016.

[8] Department of Health . Alcohol guidelines review—report from the guidelines development group to the UK chief medical officers. London: Department of Health; 2016.

[9] Health Improvement Scotland . SIGN 156—Children and young people exposed prenatally to alcohol. Edinburgh: Scottish Intercollegiate Guidelines Network; 2019.

[10] NICE . Fetal alcohol spectrum disorder—Quality standard [QS204]. London: National Institute for Health and Care Excellence; 2019.

[11] Blaylock R , Trickey H , Sanders J , Murphy C . WRISK voices: a mixed‐methods study of women's experiences of pregnancy‐related public health advice and risk messages in the UK. Midwifery. 2022;113:103433. 35878539 10.1016/j.midw.2022.103433 PMC9490559

[12] Lowe PK , Lee EJ . Advocating alcohol abstinence to pregnant women: some observations about British policy. Health Risk Soc. 2010;12:301–311.

[13] Brown R , Trickey H . Devising and communicating public health alcohol guidance for expectant and new mothers: a scoping report. London: Alcohol Concern; 2018.

[14] Lyerly AD , Mitchell LM , Armstrong EM , Harris LH , Kukla R , Kuppermann M , et al. Risk and the pregnant body. Hast Cent Rep. 2009;39:34–42. 10.1353/hcr.0.0211 PMC3640505 20050369

[15] Rosenberg G , Bauld L , Hooper L , Buykx P , Holmes J , Vohra J . New national alcohol guidelines in the UK: public awareness, understanding and behavioural intentions. J Public Health (Oxf). 2018;40:549–556. 28977621 10.1093/pubmed/fdx126 PMC6166584

[16] Schölin L , Watson J , Dyson J , Smith LA . Midwives' views on alcohol guidelines: a qualitative study of barriers and facilitators to implementation in UK antenatal care. Sex Reprod Healthc. 2021;29:100628. 33946025 10.1016/j.srhc.2021.100628

[17] Kesmodel US , Urbute A . Changes in drinking patterns, and attitudes toward and knowledge about alcohol consumption during pregnancy in a population of pregnant Danish women. Alcohol Clin Exp Res. 2019;43:1213–1219. 30908663 10.1111/acer.14031

[18] Cook M , Leggat G , Pennay A . Change over time in Australian newspaper reporting of drinking during pregnancy: a content analysis (2000‐2017). Alcohol Alcohol. 2020;55:690–697. 32676647 10.1093/alcalc/agaa072

[19] Popova S , Dozet D , Akhand Laboni S , Brower K , Temple V . Why do women consume alcohol during pregnancy or while breastfeeding? Drug Alcohol Rev. 2022;41:759–777. 34963039 10.1111/dar.13425 PMC9305227

[20] Taylor A , Whittaker A , Chandler A , Carnegie E . Accounts of women identified as drinking at ‘high risk’ during pregnancy: a meta‐ethnography of missing voices. Int J Drug Policy. 2023;117:104061. 37245246 10.1016/j.drugpo.2023.104061

[21] Bell K , McNaughton D , Salmon A . Medicine, morality and mothering: public health discourses on foetal alcohol exposure, smoking around children and childhood overnutrition. Crit Public Health. 2009;19:155–170.

[22] Rothman BK . Pregnancy, birth and risk: an introduction. Health Risk Soc. 2014;16:1–6.

[23] Burton‐Jeangros C . Surveillance of risks in everyday life: the agency of pregnant women and its limitations. Soc Theory Health. 2011;9:419–436.

[24] Lupton D . ‘Precious cargo’: foetal subjects, risk and reproductive citizenship. Crit Public Health. 2012;22:329–340.

[25] Hallgrimsdottir HK , Benner BE . ‘Knowledge is power’: risk and the moral responsibilities of the expectant mother at the turn of the twentieth century. Health Risk Soc. 2014;16:7–21.

[26] Armstrong EM , Abel EL . Fetal alcohol syndrome: the origins of a moral panic. Alcohol Alcohol. 2000;35:276–282. 10869248 10.1093/alcalc/35.3.276

[27] Mackenzie J , Zhao S . Doing motherhood online: parenting, identity and digital interaction. Discourse, context & media. 2022.

[28] Moore D , Ayers S , Drey N . A thematic analysis of stigma and disclosure for perinatal depression on an online forum. JMIR Ment Health. 2016;3:e18. 27197516 10.2196/mental.5611 PMC4909386

[29] Wigginton B , Gartner C , Rowlands IJ . Is it safe to vape? Analyzing online forums discussing e‐cigarette use during pregnancy. Womens Health Issues. 2017;27:93–99. 27773530 10.1016/j.whi.2016.09.008

[30] Toutain S . What women in France say about alcohol abstinence during pregnancy. Drug Alcohol Rev. 2010;29:184–188. 20447227 10.1111/j.1465-3362.2009.00136.x

[31] Binder A , Kilian C , Hanke S , Banabak M , Berkenhoff C , Petersen KU , et al. Stigma and self‐stigma among women within the context of the German “zero alcohol during pregnancy” recommendation: a qualitative analysis of online forums and blogs. Int J Drug Policy. 2024;124:104331. 38241887 10.1016/j.drugpo.2024.104331

[32] Binder A , Preiser C , Hanke S , Banabak M , Huber C , Petersen KU , et al. Researching alcohol consumption during pregnancy. Opportunities and challenges with two methods of data acquisition. Qual Health Res. 2022;32:1809–1827. 36017584 10.1177/10497323221119005 PMC9511243

[33] Thom B , Herring R , Milne E . Drinking in pregnancy: shifting towards the ‘Precautionary Principle’. In: MacGregor S , Thom B , editors. Risk and substance use: framing dangerous people and dangerous places. London: Routledge; 2020.

[34] Timmermans S , Tavory I . Data analysis in qualitative research: theorizing with abductive analysis. Chicago: University of Chicago Press; 2022.

[35] Tavory I , Timmermans S . Abductive analysis—theorizing qualitative research. Chicago and London: University of Chicago Press; 2014.

[36] Gee JP . An introduction to discourse analysis—theory and method. 4th ed. London: Routledge; 2014.

[37] Similarweb . mumsnet.com. 2022. Available from: https://www.similarweb.com/website/mumsnet.com/#overview

[38] British Psychological Society . Ethics guidelines for internet‐mediated research. Leicester: British Psychological Society; 2021.

[39] Harris J , Germain J , Maxwell C , Mackay S . The ethical implications of collecting data from online health communities. Sage Research Methods Cases: Medicine and Health. 2020. 10.4135/9781529716085

[40] Eysenbach G , Till JE . Ethical issues in qualitative research on internet communities. BMJ. 2001;323:1103–1105. 11701577 10.1136/bmj.323.7321.1103 PMC59687

[41] Lowe P . Milk for a girl and bananas for a boy: recipes and reasons for sex‐preference practices in a British internet forum. Women's Reprod Health. 2015;2:111–123.

[42] Kukla R . Measuring mothering. Int J Fem Approaches Bioeth. 2008;1:67–90.

[43] Hays S . Cultural contradictions of motherhood. New Haven, CT: Yale University Press; 1998.

[44] Wolf JB . Is breast best? Taking on the breastfeeding experts and the new high stakes of motherhood. New York: NYU Press; 2011.

[45] Waggoner MR . The zero trimester: pre‐pregnancy care and the politics of reproductive risk. Oakland, CA: University California Press; 2017.

[46] Meurk CS , Broom A , Adams J , Hall W , Lucke J . Factors influencing women's decisions to drink alcohol during pregnancy: findings of a qualitative study with implications for health communication. BMC Pregnancy Childbirth. 2014;14:246. 25060554 10.1186/1471-2393-14-246 PMC4122767

[47] Hammer R , Gouilhers S , Radu I , Pehlke‐Milde J , Meyer Y . Uncertainty, manageability and individuation: a longitudinal qualitative study of women's conceptualisations of risk from pregnancy to breastfeeding—the case of alcohol consumption. SSM Qual Res Health. 2022;2:100152.

[48] McDonald K , Amir LH , Davey M‐A . Maternal bodies and medicines: a commentary on risk and decision‐making of pregnant and breastfeeding women and health professionals. BMC Public Health. 2011;11:S5. 10.1186/1471-2458-11-S5-S5 PMC3247028 22168473

