# Supplementary material for: Representations of ‘risky’ drinking during pregnancy on Mumsnet: A discourse analysis
Source: Drug Alcohol Rev. 2024 Sep 19;44(1):26–36. doi: 10.1111/dar.13948 (PMC11743107; doi:10.1111/dar.13948)
Supplement: Supplementary file 1 — Table S1. Coding framework. [file DAR-44-26-s001.docx]

**Table S1. Coding framework**

| **Top Level Theme** | **Subcategory** | **Description** | **Code** | **Discourse** |
| --- | --- | --- | --- | --- |
| **Risk** | Risk of alcohol | Harm, fetal outcomes, whether a baby is okay/not okay from drinking | RFD_certain | Certainty about risk/no risk and causal relationship (regardless of amount) |
|  |  |  | RFD_uncertain | Not knowing risks/thresholds/associations for harm/FASD etc. |
|  |  |  | RFD_ minimising | Play down the risks to the fetus |
|  |  |  | RFD_maximising | Exaggerate/blow up the risks of alcohol (regardless of amount) |
|  |  |  | RFD_heavy | Heavy alcohol use causes FAS/negative consequences |
|  | Risk of low alcohol/alcohol free alternatives | Generic discussions about consuming these products | NL_definition | The formal definition of no/lo and how that links to alcohol content |
|  |  |  | NL_choice | Choosing alcohol-free rather than low alcohol to minimise, even if belief is that small amounts is likely fine or that low alcohol is fine |
|  |  |  | NL_safe | Statements about no/lo (either no or low alcohol beverages) are safe |
|  | Othering of ‘addicts’ | Distancing from ‘others’/women at risk for FASD, emphasising addiction as part of risk | OA_onlyFASD | Only women with addiction problems have babies with FASD |
|  |  |  | OA_someFASD | Not all women with addiction problems have babies with FASD |
|  |  |  | OA_OwnDrinking | Their own drinking isn’t problematic, not like “others” |
| **Evidence (outside sources)** | “Official” guidance | Discussions that explicitly mention official guidance (old/current/new etc) | OG_change | Noting or acknowledging that the guidance has changed (e.g. to justify why drinking small amount is) |
|  |  |  | OG_Postive | Positive views related to OG, indicating trusting it/the science behind it |
|  |  |  | OG_Negative | Negative views related to OG, indicating distrust in the advice/the science behind it |
|  |  |  | OG_Uncertain | Uncertainty about OG |
|  |  |  | OG_Reliance | Showing reliance on OG (i.e. not drinking because of the guidance or drinking because of the guidance under old guidance) |
|  |  |  | OG_Dismiss | Dismissing OG and adjusting behaviour accordingly (i.e. drinking because not trusting guidance) |
|  | Personal stories and anecdotes | Personal stories (own or others) as evidence of harm/risk/outcomes | PA_Positive | Sharing of stories of positive outcomes (e.g. drinking didn’t harm baby), either to reassure or provide information |
|  |  |  | PA_Negative | Stories of situations where there was a negative outcome (e.g. FASD from some level of exposure) |
|  |  |  | PA_Uncertain | Personal stories where it's not clear one way or the other what the risk is/what the outcome is, lots of doubt of validity of personal stories |
|  |  |  | PA_Reliance | Reassurance seeking etc receiving reassurance |
|  |  |  | PA_Dismiss | Dismissing PA as evidence of how to behave |
|  |  |  | PA_directing | When a poster is giving a direct advice to the OP (that is not to seek professional advice) |
|  | Academic and scientific studies | Formal evidence from books, scientific studies or unnamed scientific studies (“I read about X” without specifying where it came from) | AS_Accepting | Citing scientific evidence that proves/disproves harm from alcohol at a certain level |
|  |  |  | AS_Uncertain | Unclear what the evidence is/coming form/what is says |
|  |  |  | AS_Certain | Drawing very firm conclusions from the evidence |
|  |  |  | AS_Reliance | Relying on academic or scientific studies |
|  |  |  | AS_Critical | Being critical of scientific evidence as unreliable (e.g. because of study design, study population etc) |
|  | Advice from medical professionals (midwives etc) | Any experiences that involves health professionals (either from a conversation that has happened or people directing someone to gather information) | MP_directing | Directing the poster to seek advice from medical professionals for advice/reassurance |
|  |  |  | MP_reassuring | Advice received from medical professionals are reassuring |
|  |  |  | MP_NotReassuring | When advice from medical professionals are not reassuring |
|  |  |  | MP_guidance | Medical professionals’ direct guidance related to alcohol use or instruction (not just giving reassurance), i.e. needs to have direction |
| **Interpretation Social Norms** | Identity | Discourse that speaks to how posters see or feel about themselves | ID_positive | “Positive” view of self |
|  |  |  | ID_negative | Feeling shamed of having drunk, feeling guilt/regret |
|  |  |  | ID_tension | Tension on how poster *feels/articulates* about self/their actions in relation to how they present themselves (e.g. sounding very educated and relying on evidence but seeking reassurance from anecdotal evidence) or an implicit tension based on how they talk about their own behaviour (e.g. binge) vs. how they talk about evidence (e.g. high doses) – N.B. any use of this code should be where there are at least 2 different “identities” in the post |
|  |  |  | ID_prof | Using own profession/affiliation/occupation to emphasise point or add authority |
|  | Justifying drinking | Acknowledging that drinking, in general, is so normal that exposure before knowing or during 'acceptable' times are okay during pregnancy (specific times, amounts, types of alcohol). Related to social expectations in society (drinking in the sun, on holiday etc). | JD_Types | Justifying drinking based on types of drinks (prosecco, 'cold white wine', Shandy, cocktails etc vs hard liquor) in relation to risk |
|  |  |  | JD_Occasion | Justifying drinking based on occasion (i.e. on holiday, in the sun etc) which is embedded in wider social norms of drinking at certain occasions |
|  |  |  | JD_modifying | Reducing/modifying risk by diluting drinks |
|  |  |  | JD_pace | Pacing drinking or otherwise manage risk (e.g. by having it with meal) |
|  |  |  | JD_addiction | Justifying drinking based on addiction/alcoholism |
|  |  |  | JD_conception | Normalising drinking around conception/before knowing |
|  |  |  | JD_others | Justifying own drinking during pregnancy based on perception that it's a common occurrence |
|  |  |  | JD_choice | Choosing to drink when pregnant as an informed decision rather than/in addition to defying social norms |
|  | Social norms/expectations of behaviour during pregnancy | Explicit or implicit recognition of societal expectations or behaviours during pregnancy | SN_acknowledge | Acknowledging expectations |
|  |  |  | SN_adherence | Adhering to expectations |
|  |  |  | SN_defying | Defying expectations |
|  |  |  | SN_unsure | Uncertain about what the ‘norm is’ (whether it is not drinking or having some alcohol) and whether everyone is on the same page of appropriate behaviour |
|  |  |  | SN_cravings | Any discussion about cravings at all that also acknowledges the underlying advice of not drinking |
|  |  |  | SN_drinkculture | Comments about being all or nothing with drinking reflecting overall drinking culture and their own pre-pregnancy habits |
